# Digital support for chronic dyspnoea management in primary care: protocol for the BREATHE (Breathlessness Rapid Evaluation and Therapy) cluster randomised controlled trial

**DOI:** 10.1136/bmjopen-2025-108255

**Published:** 2025-12-31

**Authors:** Allison Martin, Anthony Paulo Sunjaya, Katrina Giskes, Zoe McKeough, Charlotte M Hespe, Clare Arnott, Laurent Billot, Anna Campain, Caroline Polak Scowcroft, Emily R Atkins, Stephen Jan, Hayley A Scott, Ai-Vee Chua, Christine R Jenkins, on behalf of the BREATHE Investigators, David Peiris

**Affiliations:** 1 The George Institute for Global Health, Sydney, New South Wales, Australia; 2 University of New South Wales, Sydney, New South Wales, Australia; 3 The University of Notre Dame Australia School of Medicine Sydney Campus, Darlinghurst, New South Wales, Australia; 4 Discipline of Physiotherapy, The University of Sydney, Sydney, New South Wales, Australia; 5 Royal Prince Alfred Hospital, Camperdown, New South Wales, Australia; 6 Consumer representative, The George Institute for Global Health, Sydney, New South Wales, Australia; 7 Priority Research Centre for Healthy Lungs, University of Newcastle School of Biomedical Sciences and Pharmacy, New Lambton Heights, New South Wales, Australia; 8 School of Rural Health, The University of Sydney, Sydney, New South Wales, Australia

**Keywords:** Primary Care, RESPIRATORY MEDICINE (see Thoracic Medicine), Cardiovascular Disease, Clinical Decision-Making

## Abstract

**Introduction:**

Chronic dyspnoea is a prevalent symptom, and primary care is ideally placed to identify and manage it. However, chronic dyspnoea is under-reported by patients and can be a diagnostic dilemma for practitioners. A fully automated system of patient screening, coupled with a clinical decision support system (CDSS) that uses a validated and evidence-based dyspnoea algorithm, may improve detection, diagnosis and management of the condition. There is currently no CDSS validated for chronic dyspnoea diagnosis and management in primary care in Australia. The objectives of this study are to assess the clinical impact of a CDSS for chronic dyspnoea in primary care. We hypothesise that the use of the CDSS will lead to a clinically significant improvement in patient-reported dyspnoea scores, reduced time to diagnosis and healthcare costs at 12 months compared with standard care.

**Methods and analysis:**

The BREATHE study is an open-label, cluster-randomised controlled trial of standard of care compared with a CDSS. General practices (n=40) in metropolitan, regional/rural and rural/remote settings will be recruited and randomised equally to pre-screening for chronic dyspnoea and usual standard-of-care management or pre-screening *and* CDSS-guided management. The CDSS includes an algorithm derived from a robust data and clinical knowledge model and incorporates evidence-based recommendations for the assessment and management of chronic dyspnoea. It is integrated into general practice medical software systems, fitting in the workflow of general practitioners (GPs). Eligible patients will be ≥18 years old and will have previously consented to receive SMS communication from their practice. In-scope patients will receive an automated text message prior to their GP appointment and will be screened for chronic dyspnoea (≥4 weeks). Patients identified with chronic dyspnoea will be invited to participate in the BREATHE study and followed up for 12 months. The primary outcome is improvement in the Dyspnoea-12 (D-12) score from baseline to 12 months, measured by the Dyspnoea-12 (D-12) questionnaire. Secondary outcomes include disease-specific questionnaires to assess changes in clinical outcomes, time to final diagnosis, quality of life, healthcare utilisation and costs incurred to patients.

**Trial registration number:**

The trial is registered at ANZCTR (ACTRN12624001451594). ANZCTR is a primary registry that meets the requirements of the ICMJE and is listed on the ICTRP Registry Network.

**Ethics and dissemination:**

The study protocol has been approved by the University of New South Wales Human Research Ethics Committee (HREC) (iRECS6645) and complies with the National Health and Medical Research Council ethical guidelines. Participating practices and each GP will provide written, informed consent. All patients being screened will provide electronic informed consent. Results of the study will be disseminated through various forums, including peer-reviewed publications and presentation at national and international conferences. Following the study, participating practices will be provided with a summary of the findings of the study, together with a full copy of any publications and a plain language statement for participants, which will be made available in the practice reception area.

**STRENGTHS AND LIMITATIONS OF THIS STUDY**

- The clinical decision support system (CDSS) for breathlessness uses an evidence-based algorithm based on routinely collected data from a large UK general practice database and is integrated within existing primary care data systems and workflow.
- The CDSS incorporates an algorithm developed using Bayesian Networks to predict twenty two conditions associated with presentations of breathlessness, ascertained by analysis of the data from 384 994 episodes of breathlessness recorded in the database. It uses probabilities to identify the highest likely cause of dyspnoea and delivers the latest evidence-based preventive care tailored for each patient depending on their history and presenting symptoms.
- The study is a pragmatic cluster randomised trial of 40 general practices including metropolitan, rural and remote sites ensuring the outcomes are representative of the Australian population.
- A current limitation is the integration with a single practice software system (Best Practice); however, this serves as a proof-of-concept, with future work planned for broader implementation.
- The use of a tool that requires SMS communication may introduce bias by excluding older, less technologically adept or non-English-speaking participants; however, using an established system within the GP workflow was vital as a proof of concept.

## Introduction

 Dyspnoea is a common presenting complaint in primary care, experienced by approximately 10% of Australians.^1^ It can be a symptom of cardiorespiratory disease, obesity, deconditioning, anxiety or a combination of factors/conditions.^2^ The development and implementation of approaches for the early and accurate detection and diagnosis of these conditions are a major unmet need. An estimated 70% of people with asthma or chronic obstructive pulmonary disease (COPD) remain undiagnosed and hence untreated in the community.^3^,^5^ Dyspnoea is complex but is often overlooked and under-recognised by patients. Research indicates that there is significant potential to support general practitioners (GPs) in enhancing screening and risk stratification for dyspnoea, helping to ensure earlier and more accurate identification of underlying conditions.^6^

GPs see at least 85% of the Australian population annually and are ideally placed to screen and manage dyspnoea; however, their provision of optimal assessment is often constrained by time pressures during patient consultations, and there are large gaps between guidelines and practice. Most GP presentations for dyspnoea are for acute episodes.^7^ Once the acute presentation has been addressed, there is often little, or no time left, to address underlying causes and management for chronic dyspnoea.

Our focus groups with GPs, specialists and allied health^8^ found that while GPs are crucial to achieving optimal care of breathless patients, the multifactorial nature of dyspnoea^2^ is a diagnostic and management challenge that limits their ability to provide the correct diagnosis or best management.^6^ New approaches are needed to reduce avoidable diagnostic tests and potentially harmful and unnecessary treatments. The gaps in care across common problems, such as asthma, COPD, cardiac failure, obesity or lifestyle issues such as deconditioning,^2^ highlight the need for integrated strategies to manage the multifactorial causes of dyspnoea^9^ in a person-centred^10^ and evidence-based way.^11^

The Breathlessness Rapid Evaluation And ThErapy (BREATHE) clinical decision support system (CDSS) is an innovative digital technology that is integrated into GP electronic medical record systems and embeds a clinical algorithm for the diagnosis and management of dyspnoea. It provides personalised treatment recommendations for the most common causes of dyspnoea identified in our previous analysis of Australian GP records,^12^ including asthma, COPD, heart failure, lower respiratory infections, pulmonary emboli and contributors such as obesity, deconditioning and anxiety.

**Figure 1:**
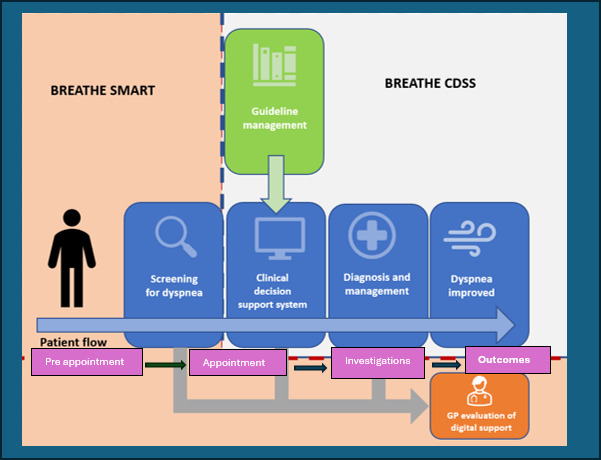
Flow of BREATHE SMART and BREATHE CDSS protocols illustrated. BREATHE, Breathlessness Rapid Evaluation And ThErapy; CDSS, clinical decision support system; GP, general practitioner.

The BREATHE CDSS study is partnering with the BREATHE SMART project, conducted by the University of Notre Dame (ACTRN12624001455550), which uses a pre-consultation screening tool as the primary mechanism to identify eligible patients with unrecognised breathlessness (see figure 1). The BREATHE SMART screening tool and the BREATHE CDSS are incorporated into a current workflow of pre-screening via a smartphone SMS (BetterConsult) which will identify eligible participants to consent to the BREATHE randomised controlled trial through the use of the modified Medical Research Council (mMRC)^13^ breathlessness scale. Once detected, a more efficient approach to incorporating evidence-based dyspnoea clinical decision support for the GPs to diagnose and manage patients is required. The use of CDSS has been shown to improve diagnosis and treatment in other diseases,^14^,^17^ although CDSS tools must be tested for their accuracy and potential for benefit over harm in each clinical context.^18^,^20^ There are very few validated dyspnoea algorithms, and none have been tested in the Australian primary care setting or integrated within the GP practice data systems or workflows.^21^ A CDSS would assist GPs in navigating the multiple possible causes of dyspnoea and deliver the latest evidence-based preventive care tailored for each patient.

The BREATHE study will use our CDSS prototype in a cluster randomised clinical trial in primary care with the aim to improve diagnostic accuracy, symptom management, clinical outcomes and diagnostic efficiency of chronic dyspnoea compared with standard clinical practices. Assessment will focus on clinical and patient-reported outcomes, clinician uptake and health economic impacts.

## Methods and analysis

### Practice recruitment

40 urban, regional and rural GP practices will be recruited across Australia with the assistance of our GP investigators and collaborators: HealthShare (BetterConsult software) and Ochre Health, and through advertising the project via newsletters from the Royal Australian College of General Practitioners (RACGP) and the Primary Health Networks and at GP meetings.

### Eligibility

General practice

1. Use BetterConsult software.^22^
2. Provide written informed consent to participate (online supplemental file 1).

All general practitioners

1. Provide written informed consent to participate (online supplemental file 2).

Participants

1. Aged ≥18 years.
2. Consent to text message (SMS) communication with their GP practice.
3. Dyspnoea lasting ≥4 weeks.
4. Provide electronic informed consent (e-consent) to participate (onlinesupplemental files 3  4).

### Intervention

The BREATHE CDSS incorporates a data- and clinical-driven algorithm for diagnosis of breathlessness: the data model embedded in the CDSS has been developed and validated through in-depth statistical analyses of a large GP database from the UK (>1000 practices)^23^ and uses a Bayesian Network^24^ approach to calculate posterior probabilities for each of the 22 potential diagnoses based on the integrated input of patient demographics, pre-screening symptom data, GP-entered physical examination findings and historical data extracted from the electronic medical record (eMR).^25^ Bayesian Network CDSS tools are gaining popularity because of their ability to be flexible with respect to disparate data and their open box approach.^26 27^ The data-driven model was overlaid with a clinical knowledge model. The 22 relevant diagnoses for breathlessness that the CDSS can detect, and the component that contributes to each, are summarised in table 1. The integration of the two components ensures that the BREATHE CDSS probabilities are clinically appropriate and supported by data. Importantly, it recommends cost and time-effective diagnostic tests and provides links to evidence-based guidelines and patient resources for each provisional diagnosis to align management with best practice guidelines and to enable GPs to provide patients with additional patient-based resources. This clinical algorithm was incorporated into an existing medical software platform already in place in a large proportion of Australian primary care networks (BetterConsult) to ensure translation into practice via existing workflows.

**Table 1:** Components of the models used in the algorithm that contribute to the diagnoses that the Breathlessness Rapid Evaluation And ThErapy clinical decision support system is trained to detect

Relevant diagnosis	Data model	Knowledge model
Anaemia		YES
Anxiety		YES
Asthma	YES	YES
Chronic bronchitis	YES	YES
COPD	YES	YES
Costochondritis		YES
Heart (other)		YES
Heart block or clinically important conduction defect		YES
Heart failure	YES	YES
ILD (pulmonary fibrosis)	YES	YES
Lung cancer	YES	YES
Myocardial infarct/heart attack		YES
Non-infective pneumonitis/infiltrate		YES
Non-pneumonia LRTI	YES	YES
Pneumonia	YES	YES
Pulmonary embolism	YES	YES
Pulmonary hypertension/right heart strain		YES
Rib fracture		YES
Tachyarrhythmia (including Atrial Fibrillation)		YES
VCD		YES
Deconditioning (loss of fitness)		YES
Dysfunctional breathing/breathing pattern disorder	YES	

COPD, chronic obstructive pulmonary disease; ILD, interstitial lung disease; LRTI, lower respiratory tract infection; VCD, vocal cord dysfunction.

### Trial design

The pragmatic implementation trial will be a cluster randomised controlled 2-arm trial (RCT) in 40 primary care sites. Each arm will comprise 20 practices, outlined below:

ARM 1: pre-screening for chronic dyspnoea and standard of care management.

ARM 2: pre-screening for chronic dyspnoea and CDSS guided management

Practices will be recruited from urban, regional and rural areas and individual GPs within each practice will consent to participate. Recruitment of patients will continue for 12 months. We will purposefully sample practices from all categories to ensure representation of each region (metropolitan, regional, rural) is equal in each arm. The randomised ‘units’ in this cluster RCT are the GP practices. The GP practices will be enrolled over time, so the randomisation list will be created by an unblinded statistician and (blinded to the study team) include a schedule within each regionality using randomly permuted blocks (a mix of size two or four) to ensure balance, and we will allocate and advise the study team as sites are activated, as GP practice allocation will be unblinded to both the study team and the GP practice.

### Participant recruitment

It is estimated that around 300 patients per practice (based on an average practice size) will undertake the pre-screening over a 12 month period, which will result in approximately a total of 12 000 patients completing the pre-screen survey. Out of these 12 000, we expect dyspnoea rates of around 10%. However, considering variations in severity/duration of breathlessness and patient consent means we are allowing for 3/4 patients not to proceed to the CDSS, resulting in a total of 400 (200 per arm) who will be identified as having chronic dyspnoea and who will consent to the BREATHE study. This means a target for each practice to recruit a minimum of 10 participants, who will be followed up for 12 months.

### Procedures

All patients who book a GP consultation will receive an SMS prior to their appointment with a standard set of questions developed by BetterConsult that is completed on their smartphone. An additional mMRC question has been added to this to identify any patients with breathlessness. Patients who indicate an mMRC ≥1 will also be asked the timeframe of their breathlessness and to answer the Dyspnea-12 (D-12) questionnaire.^28^

Patients who have an mMRC ≥1 and have been experiencing breathlessness for ≥4 weeks will be invited to participate in the BREATHE study and will complete an e-consent on their smartphone. The e-consent is an ‘easy-read’ format designed to be read on a smart phone. All consented participants will be followed up at 3, 6 and 12 months, but their intervention will differ depending on which intervention arm their GP practice is randomised to:

#### Arm 1: pre-screening for dyspnoea and standard of care management (figure 2)

**Figure 2:**
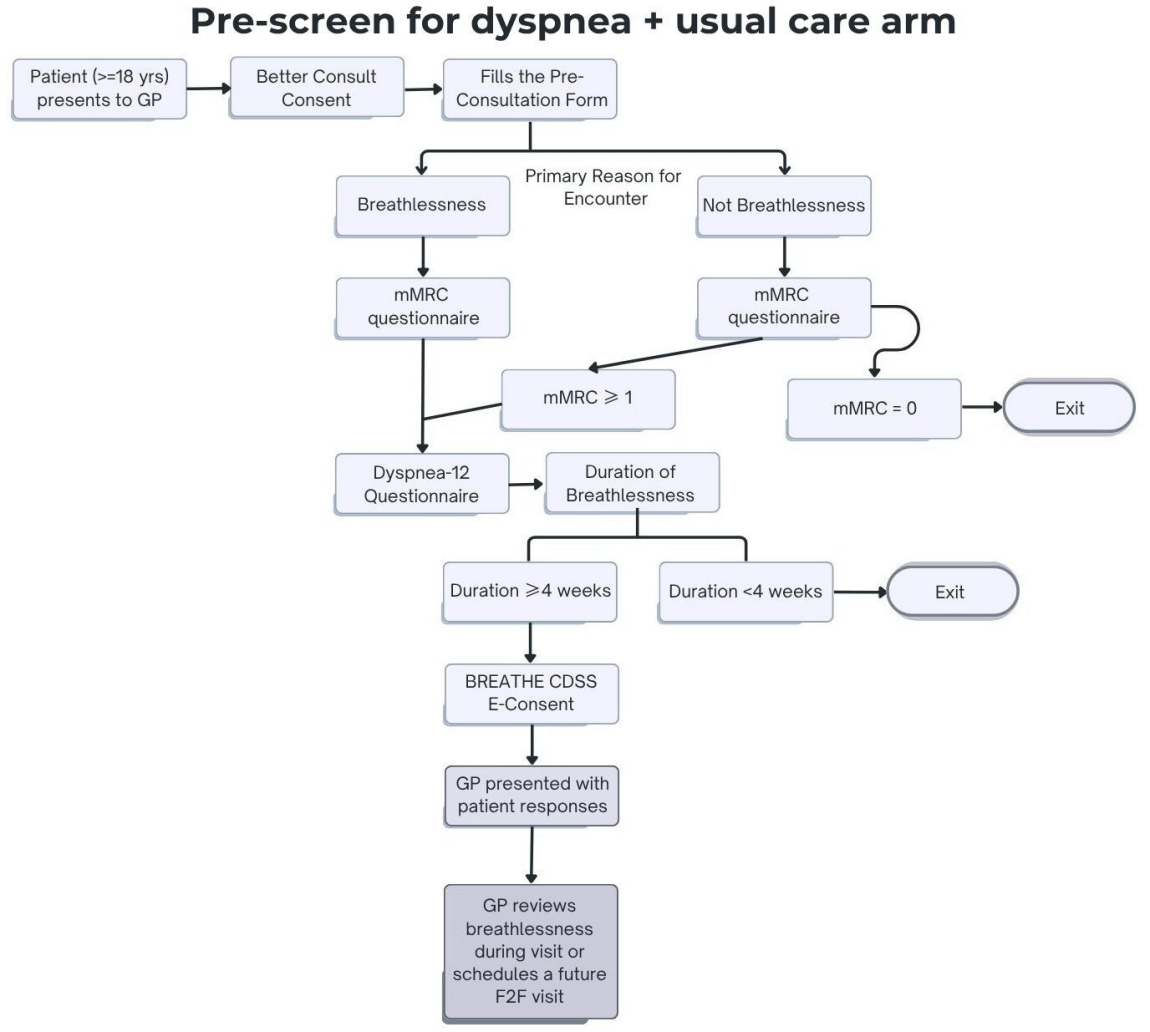
Study flowchart for Arm 1: pre-screening for dyspnoea and standard of care management. All participants at 3, 6 and 12 months will receive an SMS message to complete the following questionnaires: mMRC, D-12, EQ-5D-5L as well as have their Electronic Medical Records extracted monthly by Better Consult for the duration of the study. BREATHE, Breathlessness Rapid Evaluation And ThErapy; CDSS, clinical decision support system; GP, general practitioner; mMRC, modified Medical Research Council.

Figure 2: After obtaining consent, the participant will complete the Five-level EuroQol five-dimensional questionnaire (EQ-5D-5L)^29^ quality of life questionnaire and the Patient Health Questionnaire-4 (PHQ-4).^30^ At 3, 6 and 12 months from the date of consent, a follow-up SMS will be sent to participants to complete the mMRC, D-12, EQ-5D-5L and PHQ-4 to assess changes in breathlessness and quality of life. They will also be asked if they had any hospital admissions for the health economics evaluation. De-identified information on participant demographics, medical and smoking history, vital signs, referrals, radiological and pathology results, medications and new diagnoses will be automatically extracted directly from the GP eMR for the assessment of GP management on a regular basis during follow-up.

#### Arm 2: pre-screening for dyspnoea and BREATHE CDSS-guided management (figure 3)

**Figure 3:**
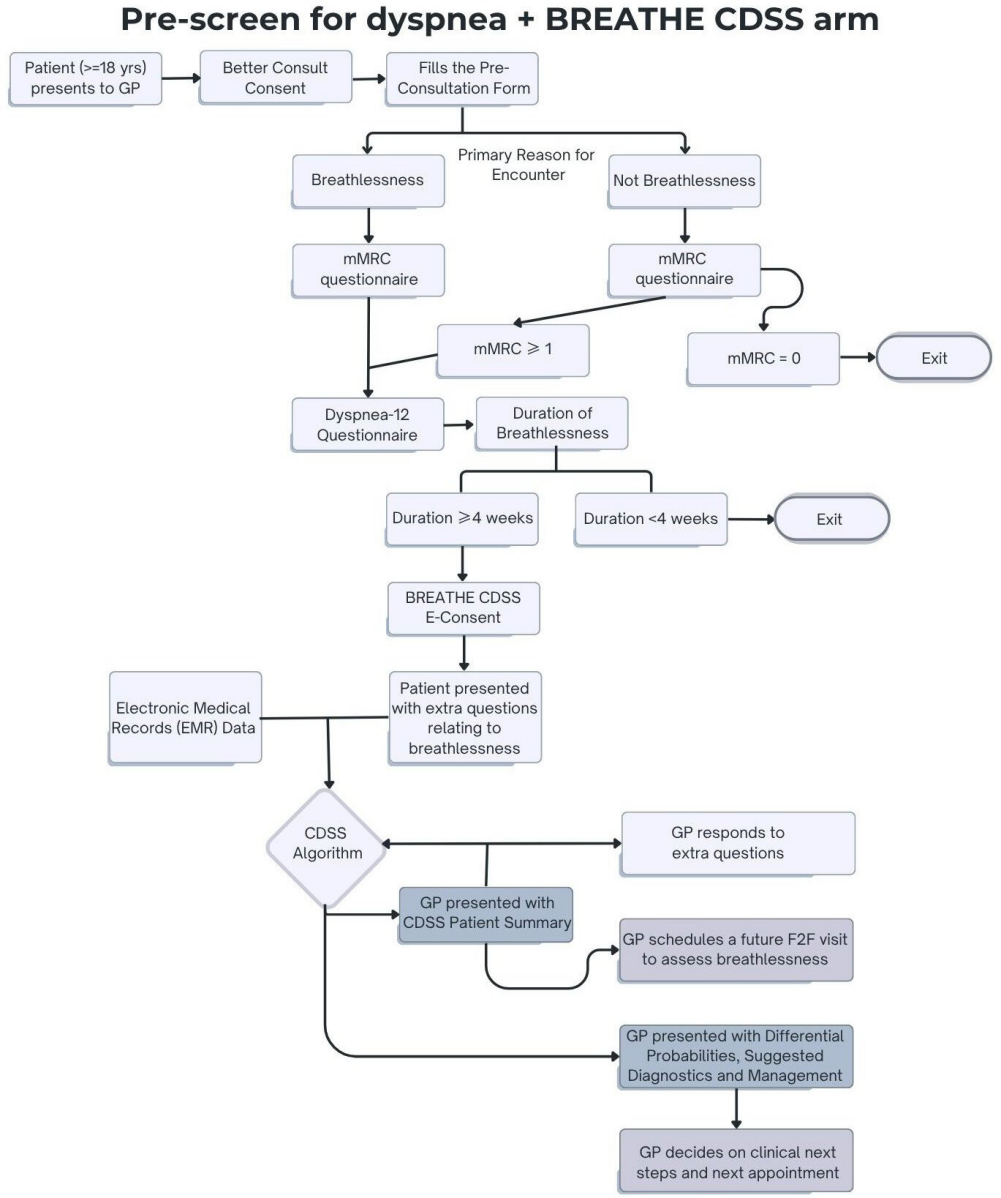
Study flowchart for Arm 2: pre-screening for dyspnoea and BREATHE CDSS intervention. All participants at 3, 6 and 12 months will receive an SMS message to complete the following questionnaires: mMRC, D-12, EQ-5D-5L as well as have their Electronic Medical Records extracted monthly by Better Consult for the duration of the study. BREATHE, Breathlessness Rapid Evaluation And ThErapy; CDSS, clinical decision support system; GP, general practitioner; mMRC, modified Medical Research Council.

Figure 3: After obtaining consent, the participant will complete additional questions about their breathlessness and the EQ-5D-5L and the PHQ-4. Responses to all questions will be forwarded to the GP for review prior to the consultation. The GP will be directed to use the BREATHE CDSS to enter more information about the participant such as vital signs and physical examination results. This information, along with the patient responses and information extracted from the eMR, will be used in the CDSS algorithm to provide probabilities for most likely diagnoses and the most relevant diagnostic tests to perform. It is expected that the GP will use this information to inform their management, but decisions will always be at the discretion of the GP. The GP will enter their provisional diagnosis into the CDSS and if they plan to refer the participant for diagnostic tests or to a specialist. For the following provisional diagnoses, the GP will ask participants to complete the disease-specific questionnaires:

1. COPD Assessment Test (CAT)^31^ for participants provisionally diagnosed with COPD.
2. Chronic Airways Assessment Test (CAAT)^32^ for participants provisionally diagnosed with asthma.
3. Kansas City Cardiomyopathy Questionnaire (KCCQ)^33^ for participants provisionally diagnosed with heart failure.
4. Nijmegen Questionnaire^34^ for participants provisionally diagnosed with dysfunctional breathing.

At all scheduled appointments from this point up until 12 months, the GP can access the CDSS to update results or information and the algorithm will recalculate the probabilities for diagnoses. At 3, 6 and 12 months, the participants will attend a clinic visit and the SMS sent prior to this appointment will include the mMRC, D-12, EQ-5D-5L and PHQ-4, relevant disease-specific questionnaire(s), and a questionnaire asking if they had any hospital admissions visits since their last visit. Also at 3, 6 and 12 months, the GPs will enter details of tests conducted and the outcomes and update their diagnoses. De-identified information on participant demographics, medical and smoking history, vital signs, referrals, radiological and pathology results, medications and new diagnoses will be automatically extracted directly from the eMR.

### Primary and secondary outcomes

The primary outcome is improvement in dyspnoea, measured by a change in D-12 score between baseline and 12 months.

The secondary outcomes will be:

- Proportion of participants reaching disease control or clinically important improvement as measured by disease specific questionnaires:
  - CAT for COPD: a decrease of ≥2 points.
  - KCCQ for heart failure: an increase of ≥5 points.
  - CAAT for asthma: a decrease of ≥2 points.
  - Nijmegen questionnaire for dysfunctional breathing: achieving a score ≥20.
- Improvement in dyspnoea, as measured by a change in D-12 score at 3 and 6 months.
- Time to final diagnosis.
- Change in mMRC dyspnoea score at 3, 6 and 12 months.
- Change in EQ-5D-5L at 3, 6 and 12 months.
- The number of hospitalisations and length of stay.
- Number of emergency department presentations.
- Change in PHQ-4 anxiety and depression score at 3, 6 and 12 months.

### Data collection

All study data will be collected directly from the BetterConsult software of participant and GP entered information, as well as the medical information and results extracted directly from the eMR. All data will be de-identified using a 64-digit encrypted ID and uploaded into a highly secure cloud-based server housed by The George Institute for Global Health (TGI). This source data will be securely stored for 15 years. Only authorised trial staff from TGI will have access to the trial dataset. The Standard Protocol Items: Recommendations for Interventional Trials reporting guidelines were used for this protocol.^35^

All data are extracted directly from the participant, GP and practice databases and therefore considered source data, so no data monitoring is required to check for data transcription errors. There are no mandated tests or study procedures outlined in the protocol; therefore, compliance monitoring with respect to the protocol and missing data is also not required. To promote participant retention, the research team will stay in close contact with practice managers who will book the follow-up appointments for the consented patients. Data monitoring will identify upcoming and overdue visits to assist with this. Completion of the disease-specific questionnaires will also be monitored for sites randomised to the CDSS intervention arm, and if missing, the research team will contact the practice manager to follow this up with the participant.

There will be monitoring for any serious adverse events that could reasonably be considered related to study procedures. The intervention is intended solely to provide non-binding suggestions, which clinicians may choose to accept or disregard at their discretion. Therefore, clinicians retain full autonomy over all decisions regarding further investigations, management and diagnosis. The training of GPs and practice managers will cover the management of adverse events and will advise that any issues, problems or patient complaints be directed to the research team. Additionally, all participants (including practices, GPs and patients) are provided with full contact details for the relevant ethics committees in the participant information statements, should a serious adverse event or concern arise that could possibly be related to trial participation. Data monitoring for missed visits or missing data in the extraction will be conducted by the study team at TGI.

### Patient and public involvement

We conducted focus groups with GPs, specialists and allied health professionals^8^ and found that while GPs are crucial to achieving optimal care of breathless patients, the multifactorial nature of dyspnoea^2^ is a diagnostic and management challenge that limits their ability to provide the correct diagnosis or best management.^6^ Focus groups with patients confirmed their tendency not to perceive dyspnoea as a problem to raise with the GPs.^36^ Development and refinement of the protocol were an iterative process in consultation with members of the steering committees, including consumers.

The BREATHE consumer panel consists of five people diagnosed with respiratory conditions or a carer of a person with a respiratory illness. The panel meets quarterly and has reviewed the protocol and participant-facing documents, as well as participating in user acceptance testing of the participant questions in the CDSS prototype on their smartphones. The consumer panel will have ongoing input into the operational conduct of the study.

### Statistical analyses

The primary outcome is change in D-12 score, which has a minimum clinically important difference of 2.83 (out of a possible score of 36).^37^ Assuming a two-arm parallel cluster trial with a baseline measure, a total of 40 GP practices (clusters) provides 90% power to detect a mean difference in D-12 of four points at 0.05 significance level. To achieve this, each practice will be expected to recruit a minimum of nine participants who will be followed up for 12 months each. Allowing for a drop-out rate of 10%, we aim to recruit 10 participants per cluster for a total of 400 participants. A conservative SD of D-12 of 15 points and individual autocorrelation (the correlation between observations from the same individual at different times) of 0.65 is assumed. These calculations are robust to values of the intra-cluster correlation coefficient up to 0.10.

The primary analysis will compare change in D-12 scores at 12 months between the CDSS and standard care arms. The analysis will be performed using a repeated-measure linear mixed model including measurements taken at baseline, 3, 6 and 12 months. The model will include the effect of the visit, the randomised group and the interaction between randomised group and visit. A random cluster effect will be used to model within-cluster correlation, and a repeated effect will be used to model within-patient correlations.

A similar approach will be used to analyse secondary outcomes with logistic regression used in place of linear regression for binary outcomes. Controlled multiple imputations^38^ will be used to assess the robustness of the primary outcome results to different missing data assumptions. Sensitivity analyses including additional covariate adjustments will also be performed. A detailed statistical analysis plan will be developed and made publicly available before any analysis.

### Economic evaluation

A within-trial cost-utility analysis will be conducted from a health system perspective to assess the cost-effectiveness of BREATHE CDSS over standard of care. Cost estimates will be based on all aspects of the intervention. Intervention costs associated with CDSS use will be determined from project financial records, including costs associated with ongoing technical support and maintenance of the CDSS. Hospitalisation costs and subsequent GP visits, referrals and tests, as reported at each follow-up visit, will be costed using Australian National Diagnosis Related Group cost weights and the Medicare Benefit Schedule (MBS), respectively. We will seek consent from all participants to access their MBS and Pharmaceutical Benefits Scheme data via Services Australia (EREC approval number RMS4167). The outcomes of interest will be differences in health-related quality of life measured using the EQ-5D-5L between CDSS and standard of care groups over a 12 month period.^39^ The latter will be converted into utility scores via published Australian value sets to determine the quality adjusted life year gain at 12 months. A joint modelling approach using multilevel modelling of both costs and effectiveness will be used to estimate the incremental cost per quality-adjusted life year gained and thus determine whether CDSS provides value for money. Finally, a budget impact analysis will be conducted to assess the fiscal impact on the Australian healthcare system with the potential implementation of the CDSS system at a nationwide level. A series of scenario analyses with varying different assumptions around costs, effects and the population of interest will be undertaken.

## Ethics and dissemination

The study protocol (version 3.0 23 Aug 2024) has been approved by the University of New South Wales (UNSW) Human Research Ethics Committee (HREC) (iRECS6645) and will comply with the National Health and Medical Research Council’s ethical guidelines. Participating practices and each GP will provide written, informed consent which will be collected by TGI. All patients being screened will provide electronic informed consent (e-consent). All protocol amendments will be submitted and approved through UNSW HREC and sent to all participating GP practices. Results of the study will be disseminated through various forums, including peer-reviewed publications and presentation at national and international conferences. Authorship of publications will follow ICMJE guidelines and the BREATHE publication policy, and no professional writers will be employed nor artificial intelligence used. Following the study, participating practices will be provided with a summary of the findings of the study, together with a full copy of any publications and a plain language statement for participants, which will be made available in the practice reception area.

## Discussion

Over 1.8 million Australians have breathlessness (dyspnoea) that chronically limits exertion and impacts quality of life. In a recent survey of over 10 000 Australian adults,^1^ 22% reported a current respiratory or heart condition, or both. Of those with a current respiratory condition, 38% were not taking medications for a breathing or lung problem. Similarly, 41% of participants who reported a current heart condition were not taking cardiac medications, even though a quarter of these had dyspnoea that limited their daily activity and significantly reduced their productivity.^40^ Respiratory conditions account for around 10% of deaths in Australia,^3^ and almost one-third (31%) of Australian adults have a chronic respiratory disease,^1^ with a high rate of multimorbidity in this group.^41^ Unfortunately, an estimated 70% of people with asthma or COPD go undiagnosed.^3^,^5^ Cardiovascular disease accounts for 568 000 hospitalisations per year,^42^ kills one in every four Australians^43^ and co-exists with respiratory diseases, especially in the older population.^41^ Dyspnoea is a cardinal symptom of cardiovascular disease and chronic respiratory disease, and both are major health issues and among the largest contributors to life expectancy inequalities in Australia.^44^

Dyspnoea as a symptom is compounded by the multiple conditions that may co-exist having multiplicative effects. Obesity, for instance, causes a four-fold increased risk of being significantly breathless and is increasingly prevalent in both younger and older Australians.^45^ Additional contributions to the prevalence of dyspnoea in Australia include worsening of air pollution following bushfires^46^ and the SARS-CoV-2 pandemic.^47^

Preventive healthcare is cost-effective,^48^ but the time spent on preventive health is a limited and precious resource in general practice and is diminishing due to patients’ increased complexity, workforce shortages and fee-for-service funding. Secondary prevention entails earlier diagnosis and treatment of symptoms which may otherwise progress and result in poor treatment responses, increased health resource use, hospitalisation and death. Effective and efficient management of dyspnoea in primary care is important due to its increasing prevalence and impact in Australia,^3^ and substantial evidence that respiratory symptoms are poorly investigated and sub-optimally managed at present.^1 47 49^ A more consistent approach to screening dyspnoea and efficient and effective diagnosis will lead to better health outcomes.

Many interventions in general practice fail to bring about any real changes in patients’ health, as their implementation contributes to worsening time pressures for practitioners^50^ and is burdensome on practice staff. The workflow in general practice is fast-paced, and for an innovation to be successful, it must be placed within existing workflows and not add to the time or workload burden of staff. Using a tool that requires SMS communication may introduce bias by excluding older, less technologically adept or non-English speaking participants. However, having a fully integrated system of dyspnoea screening, diagnosis support and access to evidence-based guidelines and patient resources that is incorporated within existing general practice medical software systems, electronic medical records and patient workflows is a completely new approach for improving patient outcomes in primary care.

The BREATHE study will be the first CDSS for dyspnoea globally and aims to assist GPs to navigate the multifaceted causes of dyspnoea and efficiently diagnose and treat affected patients.

## Supplementary material

10.1136/bmjopen-2025-108255 online supplemental file 1

10.1136/bmjopen-2025-108255 online supplemental file 2

10.1136/bmjopen-2025-108255 online supplemental file 3

10.1136/bmjopen-2025-108255 online supplemental file 4
