# Supplementary material for: Digital support for chronic dyspnoea management in primary care: protocol for the BREATHE (Breathlessness Rapid Evaluation and Therapy) cluster randomised controlled trial
Source: BMJ Open. 2025 Dec 31;15(12):e108255. doi: 10.1136/bmjopen-2025-108255 (PMC13059914; doi:10.1136/bmjopen-2025-108255)

BREATHE e-consent prototype for patient participants

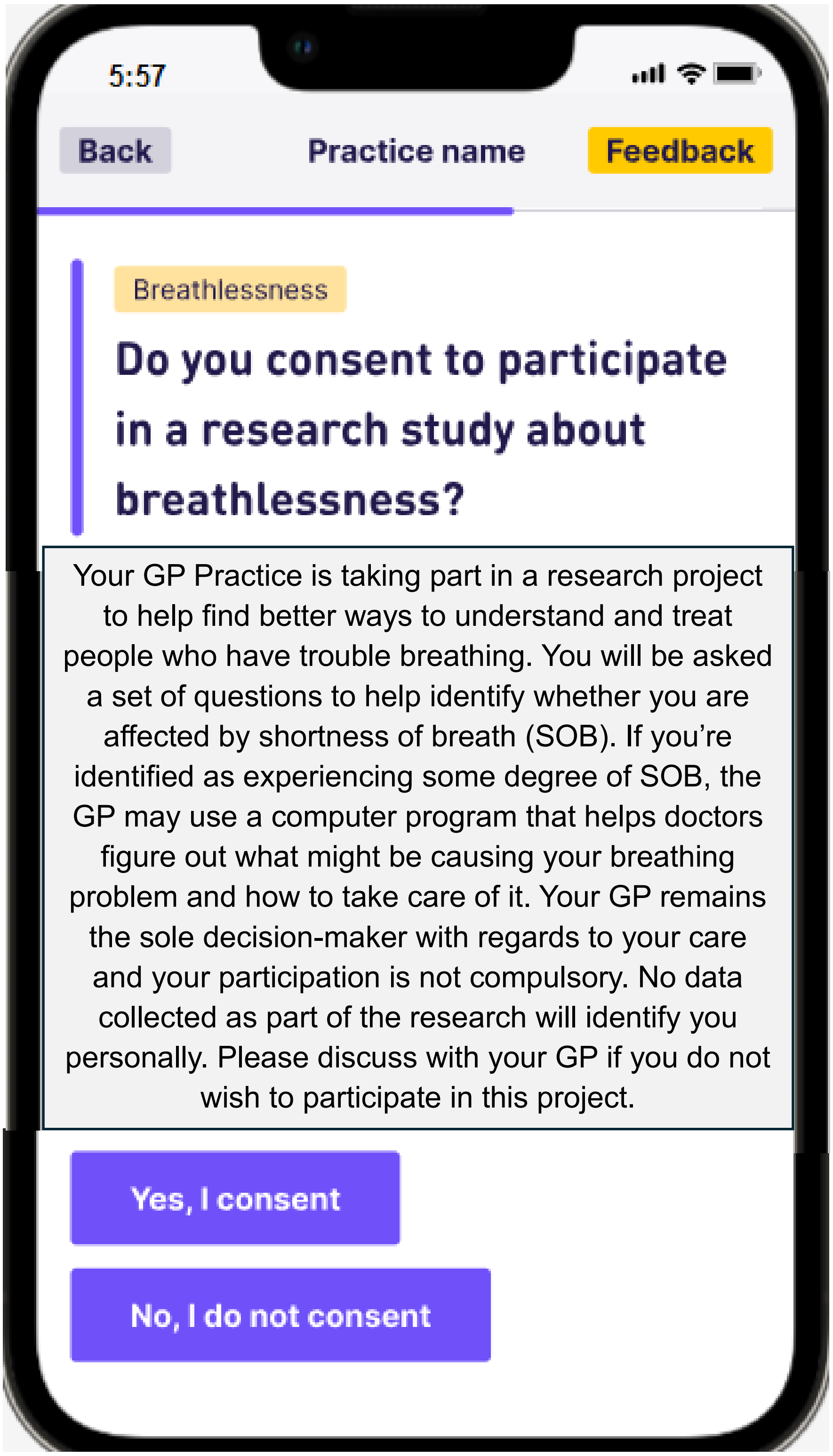

Supplement: online supplemental file 1 [file bmjopen-15-12-s001.pdf]
