# Supplementary material for: Digital support for chronic dyspnoea management in primary care: protocol for the BREATHE (Breathlessness Rapid Evaluation and Therapy) cluster randomised controlled trial
Source: BMJ Open. 2025 Dec 31;15(12):e108255. doi: 10.1136/bmjopen-2025-108255 (PMC13059914; doi:10.1136/bmjopen-2025-108255)
Supplement: online supplemental file 2 [file bmjopen-15-12-s002.pdf]

The George Institute for Global Health	THE UNIVERSITY OF NOTRE DAME AUSTRALIA

GENERAL PRACTICE INFORMATION STATEMENT AND CONSENT FORM

BREATHE – The Breathlessness Rapid Evaluation And THErapy study

**Project Sponsor**	The George Institute for Global Health, UNSW and,<br>The University of Notre Dame Australia.
**Chief Investigators**	Professor Charlotte Hespe (BREATHE SMART)<br>Professor Christine Jenkins (BREATHE CDSS)

1. What is the research study about?

Your practice is invited to take part in the BREATHE (Breathlessness Rapid Evaluation And THErapy) study that aims to test an automated system to improve the accuracy and efficiency of investigating and managing patients with breathlessness. It will use a pre-consultation screening tool (BREATHE SMART) and an integrated clinical decision support system (BREATHE CDSS) in the electronic health record to support general practitioners (GPs) to better assess patients with breathlessness in primary care. Each practice will recruit ten patients over 12 months and follow-up consented patients for 12 months. The study will assess the benefits for patients and GPs in the efficiency of reaching a diagnosis, commencing evidence-based management and achieving relief of breathlessness.

2. Who is conducting this research?

The study is being carried out by a team of researchers at The George Institute for Global Health, UNSW and The University of Notre Dame Australia. The chief investigators are Professor Christine Jenkins and Professor Charlotte Hespe. Professor Christine Jenkins is the head of respiratory trials at The George Institute for Global Health, Professor of Respiratory Medicine at UNSW Sydney and a Clinical Professor in the Concord Clinical School, University of Sydney. Professor Charlotte Hespe is the Head of General Practice and Primary Care Research at the School of Medicine, University of Notre Dame, and a GP and owner of Glebe Family Medical Practice in NSW and the Chair of NSW/ACT Faculty and Director of RACGP Board from 2017-2023.

Research Funder: This research is funded through two grants awarded by the Medical Research Future Fund (MRFF); (1) the Preventive and Public Health Research Initiative for Chronic Respiratory Conditions (BREATHE CDSS) and (2) the MRFF Clinician Researchers – Applied Research in Health (BREATHE SMART).

3. Inclusion/Exclusion Criteria

The research study is looking to recruit general practices that meet all the following criteria:

- Use Best Practice platform for their electronic health record.
- The practice GPs are willing to recruit 10 participants per practice over a 12-month period.

4. What does participation in this research require, and are there any risks involved?

This is a cluster randomised clinical trial whereby your clinic will be randomised to one of two arms:

1. BREATHE SMART pre-screening tool for all in-scope patients with a GP consultation (either face-to-face consultation or telehealth consultation) followed by standard of care (usual care) for diagnosis and management of patients presenting with chronic breathlessness

2. BREATHE SMART pre-screening tool for all in-scope patients followed by BREATHE CDSS for the diagnosis of patient presenting with chronic breathlessness

This means that patients recruited at your practice will undertake pre-screening and those identified with chronic breathlessness (lasting ≥ 4 weeks) will follow whichever of these arms your practice is randomised to. After your practice consents to participate, we will train all consenting GPs in using

the pre-screener and CDSS according to the arm your clinic is randomised. We will provide an operational manual to assist with any trial specific procedures and provide support as needed throughout the study. We have co-designed the BREATHESMART Pre-screening tool and the BREATHE CDSS with GPs in order to make them as seamless as possible with GP workflow. We do not anticipate they will add to the practice workload as their intention is to add efficiency. The specific procedures and information collected during the research is detailed in the individual GP consent forms that all participating GPs in your clinic will be asked to sign.

Risks

The BREATHE SMART and BREATHE CDSS identify patients at risk and assist with diagnosing the cause of breathlessness in your patients. However, at all times patient management is controlled by their treating clinician. The systems have been developed as an aid to clinical decision making but does not override GP experience and decision making. Therefore, there should be no additional risks to patients for using the systems developed.

Additional Costs and Reimbursement

The practice will be paid for its participation in the research as reimbursement for the time in participating, but no payments will be made to individual GPs. There are no costs associated with using Better Consult software or the set-up of the integration with Best Practice. Details on practice payments are detailed in the Clinical Trial Research Agreement.

5. What will happen to information collected about the clinic, GPs and patients?

By signing the consent form, your practice consents to the research team collecting and using information specifically and only for the purposes of the research study. The de-identified data collected will:

- Be stored for a minimum of 15 years after the publication of research results;
- Include de-identified information such as test results, questionnaire responses or referral letters.
- Be uploaded into an encrypted secure cloud-based folder with strict security access by Better Consult and then automatically or manually entered by restricted study personnel into The George Institute for Global Health, UNSW and University of Notre Dame databases.
- Access to the databases will be password protected and access will be restricted to The George Institute, UNSW or the University of Notre Dame Australia BREATHE operational teams.
- After 15 years, the data will be destroyed. Computer files will be permanently deleted from computers, hard-drives, and any other locations at study completion.

6. What if I have a complaint or any concerns about the research study and will I receive compensation if suffer any injuries or have complications?

If you have any complaints or concerns about the research study, you should contact the study team as soon as possible and you will be assisted.

Complaints Contact

If you have a complaint regarding any aspect of the study or the way it is being conducted, please contact the UNSW Human Ethics Coordinator:

Position	UNSW Human Research Ethics Coordinator
Telephone	+61 2 9385 6222
Email	humanethics@unsw.edu.au
HC Reference Number	iRECS6645

7. What should we do if we have further questions about our involvement in the research study?
If you require further information regarding this study or if you have any problems which may be related to your involvement in the study, you can contact the following member/s of the research team:

Research Team Contact Details

**Name**	Dr. Allison Humphries
**Position**	Senior Research Fellow, Respiratory Programme. The George Institute for Global Health.
**Telephone**	+61 2 8052 4383
**Email**	ahumphries@georgeinstitute.org.au
**Name**	Dr Katrina Giskes
**Position**	Primary Care Research Project Manager, School of Medicine Sydney. The University of Notre Dame
**Telephone**	+61 2 8204 4698
**Email**	katrina.giskes@nd.edu.au

Chief Investigators

**Name**	Professor Christine Jenkins
**Position**	Head of Respiratory Program at The George Institute for Global Health; Professor of Respiratory Medicine at UNSW Sydney and Clinical Professor in the Concord Clinical School, University of Sydney.
**Telephone**	+61 2 8052 4465
**Email**	cjenkins@georgeinstitute.org.au
**Name**	Professor Charlotte Hespe
**Position**	Head of General Practice and Primary Care Research at the School of Medicine at the University of Notre Dame, Sydney, a General Practitioner and owner of Glebe Family Medical Practice, NSW
**Telephone**	+61 2 8204 4450
**Email**	charlotte.hespe@nd.edu.au

Practice Consent Form

I, ...[PRINT NAME], give consent for .... [GENERAL PRACTICE NAME] to participate in the research project.

Declaration by the General Practice

- ☐ We understand we are being asked to provide consent to participate in this research study;
- ☐ We have read the Participant Information Sheet;
- ☐ We understand the purposes, study tasks and risks of the research described in the study;
- ☐ We understand that the researchers may contact our staff for an interview about their experience with using BREATHE SMART and the BREATHE CDSS and that this is recorded and voluntary.
- ☐ We do not have any objections to the data being kept at the end of the study for 15 years,
- ☐ We understand that we will be given a signed copy of this document to keep.

Name of General Practice	
Name of person signing on behalf of the General Practice (please print)	
Position of person signing on behalf of the General Practice	
Signature of Position of person signing on behalf of the General Practice	
Date	

Practice Withdrawal of Participation Form

I,……………………[PRINT NAME] wish to **WITHDRAW** my consent for………………………[GENERAL PRACTICE NAME] to participate in this research study described above and understand that such withdrawal **WILL NOT** affect my relationship with The University of New South Wales, The University of Notre Dame and The George Institute for Global Health.

- ☐ I am withdrawing my consent and I would like any identifiable information collected about me which I have provided for the purpose of this research study withdrawn.
- ☐ I am withdrawing my consent to participate in further components of this research and provide my permission for the research team to retain and/or use information collected about me which I have provided for the purpose of this research.
- ☐ I am withdrawing my consent and I understand that any information already published and/or not linked to my identity cannot be withdrawn from the research.

Participant Signature

Name of Participant (please print)	
Signature of Research Participant	
Date	

The section for Withdrawal of Participation should be forwarded to:

CI Name:	Dr Allison Humphries
Email:	ahumphries@georgeinstitute.org.au
