# Supplementary material for: Digital support for chronic dyspnoea management in primary care: protocol for the BREATHE (Breathlessness Rapid Evaluation and Therapy) cluster randomised controlled trial
Source: BMJ Open. 2025 Dec 31;15(12):e108255. doi: 10.1136/bmjopen-2025-108255 (PMC13059914; doi:10.1136/bmjopen-2025-108255)
Supplement: online supplemental file 3 [file bmjopen-15-12-s003.pdf]

PARTICIPANT INFORMATION STATEMENT AND CONSENT FORM

BREATHE – The Breathlessness Rapid Evaluation And THErapy study

**Project Sponsor**	The George Institute for Global Health, UNSW and The University of Notre Dame Australia.
**Chief Investigators**	Professor Charlotte Hespe (BREATHE SMART)<br>Professor Christine Jenkins (BREATHE CDSS)

1. What is the research study about?

You are invited to take part in the BREATHE (**Br**eathlessness Rapid **E**valuation **A**nd **THE**rapy) study that aims to improve the accuracy and efficiency of investigating and managing patients with breathlessness. It will use a pre-consultation screening tool (BREATHE SMART) and an and integrated clinical decision support system (BREATHE CDSS) in the electronic health record to support general practitioners (GPs) to better assess patients with breathlessness in primary care. Each patient will participate for 12 months. The study will assess the benefits for patients and GPs in the efficiency of reaching a diagnosis, commencing evidence-based management and achieving relief of breathlessness.

The BREATHE SMART and BREATHE CDSS system will

- identify patients with breathlessness, conduct a pre-screening questionnaire via their phones or tablet, and automatically integrate these results into GP software ahead of the consultation
- Provide an electronic CDSS that works seamlessly within the practice software to provide recommendations to the GP for diagnosis and management.

We hypothesise that this will provide electronic management support to the GP prior to the patient entering the consultation room and improve diagnostic accuracy and enhance evidence-based management of breathlessness.

2. Who is conducting this research?

The study is being carried out by a team of researchers at The George Institute for Global Health, UNSW and The University of Notre Dame Australia. The chief investigators are Professor Christine Jenkins and Professor Charlotte Hespe. Professor Christine Jenkins is the head of respiratory trials at The George Institute for Global Health, Professor of Respiratory Medicine at UNSW Sydney and a Clinical Professor in the Concord Clinical School, University of Sydney. Professor Charlotte Hespe is the Head of General Practice and Primary Care Research at the School of Medicine, University of Notre Dame, and a GP and owner of Glebe Family Medical Practice in NSW and the Chair of NSW/ACT Faculty and Director of RACGP Board from 2017-2023.

Research Funder: This research is funded by two grants awarded by the Medical Research Future Fund (MRFF) – (1) the Preventive and Public Health Research Initiative for Chronic Respiratory Conditions (BREATHE CDSS) and (2) the MRFF Clinician Researchers - Applied Research in Health (BREATHE SMART).

3. Inclusion/Exclusion Criteria

In this study we will recruit 40 GP practices from urban, regional and rural areas that use Best Practice as their practice software. Within each practice a minimum of 10 patients presenting with chronic breathlessness will be recruited over a 12-month period into the BREATHE CDSS trial, to be followed up over 12 months.

It is expected that as the treating clinician, you agree to follow the procedures of the research at the initial visit and also conduct follow-up at 3, 6 and 12 months for all patients deemed eligible for the BREATHE CDSS trial. These visits may occur through an in-person or telehealth consultation.

4. Do I have to take part in this research study?

Participation in this research study is voluntary. If you do not want to take part, you do not have to. If you decide to take part and later change your mind, you are free to withdraw from the study at any stage.

If you decide you want to take part in the research study, you will be asked to:

- Read the information carefully (ask questions if necessary).
- Sign and return the consent form if you decide to participate in the study.
- Take a copy of this form with you to keep.

5. What does participation in this research require, and are there any risks involved?

Randomisation

This is a Cluster randomised clinical trial whereby your GP practice will be randomised to one of two arms:

1. BREATHE SMART pre-screening tool + standard of care (usual care) for the investigation, diagnosis and management of breathlessness

2. BREATHE SMART pre-screening tool + BREATHE CDSS for the investigation, diagnosis and management of breathlessness

This means that all patients with breathlessness (lasting ≥ 4 weeks) recruited at your practice will follow whichever of these arms your practice is randomised to. After you consent to participate, you will receive training for the processes specific to the arm your clinic is randomised to. We will provide an operational manual and support to assist with any trial-specific issues as needed throughout the study. You will be encouraged to enrol participants at their first relevant visit and follow them according to the study protocol for the next 12 months.

If you agree to participate you will be asked to complete the following procedures:

Pre-Screening

All patients booked for practice visits at your clinic will receive a pre-screening set of questions via Better Consult software via SMS. Patients can attend the pre-screening visit face-to-face or via telehealth. If, within this set of questions, the patient identifies themselves as experiencing breathlessness, they will be asked further questions specific to this symptom. This pre-consultation tool captures patients' presenting symptoms and other relevant clinical information and transfers the data into concise medical notes for GPs to review. If the patient confirms they have suffered breathlessness for ≥ 4 weeks the GP will either manage the patient as per standard of care or utilise the CDSS clinical algorithm to assist with initial testing, reaching a diagnosis and a treatment plan, depending on the study arm your practice is randomised to. The pre-screening questionnaire information is uploaded, copied, and pasted into the patient's eMR in Best Practice.

The information gathered from the patient or directly from the eMR that is utilised in the CDSS is summarised below

- Demographics & Anthropometrics
- Medical History relevant to breathlessness
- Smoking History
- Blood pressure
- Heart rate
- Questionnaires: Modified Medical Research Council (mMRC); Dyspnea-12 (D-12); Visual Analog Scale for Breathlessness (VAS); Quality of life (EQ-5D-5L)

- We will ask patients to complete additional questionnaires determined by their likely diagnosis - e.g. Asthma Control Test (ACT), COPD Assessment test (CAT), Kansas City Cardiomyopathy questionnaire (KCCQ) for heart failure, Hospital Anxiety and Depression Scale (HADS), Nijmegen questionnaire for dysfunctional breathing questionnaire.

Intervention

Once the patient has been identified and consented in the study, we will collect the following information from each subsequent visit for breathlessness if collected or updated in the eMR:

1. Tests conducted e.g. pathology, radiology, spirometry, and their results
2. Whether a referral is made to specialists
3. Updated diagnoses
4. New medications

The uploaded test results will be incorporated into the CDSS and may confirm or modify the most likely diagnosis and differential diagnoses. Further tests will be recommended when there is as yet no final diagnosis but will also enable the GP to make an independent decision about all aspects of the patient's care. Follow-up GP appointments with patients will be scheduled at 3, 6 and 12 months after their initial presentation, which can either be face-to-face or telehealth. Prior to their appointments, patients will be asked to complete the following questionnaires which will be sent via text to the patients' phone for completion. Patients' responses to all questionnaires will be made available to you at the time of consultation.

1. mMRC score for breathlessness, Dyspnea-12, and health status measurement (EQ-5D-5L)
2. A patient questionnaire for any hospital visits and follow-up smoking status.

Risks

The BREATHE SMART and BREATHE CDSS aim to identify patients with breathlessness and assist in its diagnosis and management. However, at all times patient management decisions are controlled by you as their treating clinician. The BREATHE pre-screening tool and CDSS have been developed as an aid to clinical decision making, but they do not override your experience and decision making. There should be no additional risks to yourself or your patients for using these tools.

Additional Costs and Reimbursement:

Your practice will be paid for their participation in the research as reimbursement for the time in participating, but no payments will be made to individual GPs. There are no costs associated with using Better Consult software or the set-up of the integration with Best Practice.

6. **What are the possible benefits of taking part?**
We cannot guarantee or promise that you and/or your general practice will receive any benefits from this research. However, the BREATHE SMART and BREATHE CDSS studies are designed to improve the accuracy and efficiency of investigating and managing patients with breathlessness. Possible benefits may include efficiencies in reaching an accurate diagnosis of breathlessness, initiating more targeted tests and referrals, and having access to clinician and patient resources for optimal care.

7. **What are the alternatives to taking part in the research?**
If you decide not to participate in this study, there will be no change to your relationship with your employer or organisations involved in the research.

8. What will happen to information about me?

By signing the consent form, you consent to the research team collecting and using information about your patients who participate in the study, extracted from eMR and directly entered questionnaire results.

The research team will store the de-identified data collected for this research project as required for Good Clinical Research Practice, for:

- A minimum of 15 years after the publication of research results;
- The data collected such as test results, questionnaire responses or referral letters will be de-identified on-site using the GP practice software (Better Consult) then uploaded into an encrypted secure cloud-based folder with strict security access and/or manually entered by delegated study personnel into The George Institute for Global Health database.
- Access to the data will be password protected and access will be restricted to The George Institute, UNSW and the University of Notre Dame Australia BREATHE operational teams.
- After 15 years, the stored data will be destroyed. Computer files will be permanently deleted from computers, hard-drives, and any other locations at study completion.

The information we obtain is personal information for the purposes of the Privacy and Personal Information Protection Act 1998 (NSW). You have the right to access personal information held about you by the University, the right to request correction and amendment of it, and the right to make a complaint about a breach of the Information Protection Principles as contained in the PPIP Act. Further information on how the University protects personal information is available in the UNSW Privacy Management Plan.

9. How and when will I find out what the results of the research study are?

The research team intend to publish the results of the research. All Information will be published in a way that will not identify you or your patients. At trial completion we will provide copies of the results to you and your clinic, as well as a plain language statement for any patients recruited into the study.

10. What if I want to withdraw from the research study?

If you do consent to participate, you may withdraw at any time. You can do so by notifying the BREATHE operational team and completing the 'Withdrawal of Consent Form' which is provided at the end of this document. Your decision not to participate or to withdraw from the study will not affect your relationship with the research team, The George Institute for Global Health, UNSW or The University of Notre Dame Australia. All other information collected up to the time you withdraw will be retained and used in analyses. This is important to ensure that the results of the research study can be measured properly and comply with the law. If you do not want them to do this, you must tell them before you join the research study.

Complaints Contact

If you have a complaint regarding any aspect of the study or the way it is being conducted, please contact the UNSW Human Ethics Coordinator:

**Position**	UNSW Human Research Ethics Coordinator
**Telephone**	+ 61 2 9385 6222
**Email**	humanethics@unsw.edu.au
**HC Reference Number**	iRECS6645

11. What should I do if I have further questions about my involvement in the research study?

The person you may need to contact will depend on the nature of your query. If you require further information regarding this study or if you have any problems which may be related to your involvement in the study, you can contact the following member/s of the research team:

Research Team Contact Details

**Name**	Dr. Allison Humphries
**Position**	Senior Research Fellow, Respiratory Programme. The George Institute for Global Health.
**Telephone**	+61 2 8052 4383
**Email**	ahumphries@georgeinstitute.org.au
**Name**	Dr Katrina Giskes
**Position**	Primary Care Research Project Manager, School of Medicine Sydney. The University of Notre Dame
**Telephone**	+61 2 8204 4698
**Email**	katrina.giskes@nd.edu.au

Chief Investigators

**Name**	Professor Christine Jenkins
**Position**	Head of Respiratory Program at The George Institute for Global Health; Professor of Respiratory Medicine at UNSW Sydney and Clinical Professor in the Concord Clinical School, University of Sydney.
**Telephone**	+61 2 8052 4465
**Email**	cjenkins@georgeinstitute.org.au
**Name**	Professor Charlotte Hespe
**Position**	Head of General Practice and Primary Care Research at the School of Medicine at the University of Notre Dame, Sydney, a General Practitioner and owner of Glebe Family Medical Practice, NSW
**Telephone**	+61 28204 4450
**Email**	charlotte.hespe@nd.edu.au

Consent Form – General Practitioner providing own consent

Declaration by the General Practitioner

- ☐ I understand I am being asked to provide consent to participate in this research study;
- ☐ I have read the Participant Information Sheet;
- ☐ I understand the purposes, study tasks and risks of the research described in the study;
- ☐ I provide my consent for the information collected to be used for the purpose of this research study only.
- ☐ I do not have any objections to the data being kept at the end of the study for 15 years,
- ☐ I have had an opportunity to ask questions and I am satisfied with the answers I have received;
- ☐ I understand that I am free to withdraw at any time during the study and withdrawal will not affect my relationship with any of the named organisations and/or research team members;
- ☐ I understand that I will be given a signed copy of this document to keep.

Participant Signature

Name of General Practitioner (please print)	
Signature of General Practitioner	
Date	

Form for Withdrawal of Participation

I wish to **WITHDRAW** my consent to participate in this research study described above and understand that such withdrawal **WILL NOT** affect my relationship with The George Institute for Global Health, UNSW or The University of Notre Dame Australia.

(Please tick the relevant box below regarding the data obtained)

- ☐ I am withdrawing my consent and I would like all information collected about me which I have provided for the purpose of this research study withdrawn.
- ☐ I am withdrawing my consent to participate in further components of this research and provide my permission for the research team to retain and/or use information collected about me which I have provided for the purpose of this research to date.

Please note that any information already published and/or not linked to you identity cannot be withdrawn from the research.

Participant Signature

Name of General Practitioner (please print)	
Signature of General Practitioner	
Date	

The section for Withdrawal of Participation should be forwarded to:

CI Name:	Dr Allison Humphries
Email:	ahumphries@georgeinstitute.org.au
