# Supplementary material for: Digital support for chronic dyspnoea management in primary care: protocol for the BREATHE (Breathlessness Rapid Evaluation and Therapy) cluster randomised controlled trial
Source: BMJ Open. 2025 Dec 31;15(12):e108255. doi: 10.1136/bmjopen-2025-108255 (PMC13059914; doi:10.1136/bmjopen-2025-108255)

Improving the accuracy and efficiency of assessing and managing patients with breathlessness in primary care

BREATHE, a real-world open label, cluster-randomised controlled trial

BREATHE
The Breathlessness Rapid Evaluation And THErapy study

This is an information sheet about a research trial called BREATHE

What is the research study about?

Breathlessness is a common problem, affecting around 10% of Australians adults. The BREATHE study aims to test a system to support your doctor to better identify and diagnose the cause of breathlessness. The study will assess a pre-consultation screening tool (BREATHE SMART) to identify who is breathless and an integrated clinical decision support system (BREATHE CDSS) to better diagnose the cause.

Who is conducting this research?

The Chief Investigators are Professor Christine Jenkins, Head of Respiratory, The George Institute for Global Health, UNSW and Professor Charlotte Hespe, Head of General Practice and Primary Care Research, University of Notre Dame. The research is funded by an Australian government grant called the Medical Research Future Fund (MRFF).

Inclusion/Exclusion Criteria

To be eligible for the study:

- You need to be 18 years or over.
- To participate in the BREATHE CDSS study, you will need have experienced breathlessness for 4 weeks or longer.
- Consent to SMS communication with your GP practice.

Do I have to take part in this research study?

Your participation in this research study is voluntary. If you do not want to take part, please discuss with your GP. If you decide to take part and later change your mind, you are free to withdraw from the study at any stage by contacting your GP or simply by not responding to the SMS when you receive it prior to your next scheduled GP appointment for the BREATHE study.

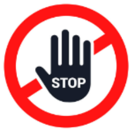

What does taking part involve?

If you are identified as experiencing some degree of shortness of breath (BREATHE SMART) you will be asked a series of questions. If you have experienced breathlessness for more than four weeks you will be managed by your GP in one of two ways. The GP practice you are attending will be assigned to either:

- Usual care with your GP or
- BREATHE CDSS intervention

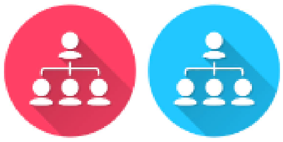

The study will follow up your health management for 12 months

If you participate:

- You will be automatically sent an SMS message to complete a list of questions on your mobile phone that are relevant to the study about your breathlessness and its possible causes which will take around 10 minutes to complete.
- If your breathlessness is chronic, you will be asked to complete more questions and see the doctor at 3, 6 and 12 months after the initial appointment to see if your breathlessness has changed. These visits will take 15 minutes and can be conducted face to face or via Telehealth. You will not be charged for these additional 3 visits.

BREATHE PIS Version 6.0_15Oct2024
https://www.georgeinstitute.org/projects/breathe
iRECS6645

The George Institute for Global Health

THE UNIVERSITY OF NOTRE DAME AUSTRALIA

Are there any risks involved?

The BREATHE CDSS intervention offers guidance to your GP, but at all times your GP is in control of your health management. It may take you extra time to complete the questions for the first visit and attend the 3 additional visits. There are no specific risks related to the pre-consultation screening tool or the clinical decision support system to better diagnose the cause of your breathlessness.

Additional Costs and Reimbursement

There are no additional costs associated with participating in this project, nor will you be paid. Your GP consultation at screening is considered part of routine care and will be subject to normal GP fees and Medicare reimbursement. The consultations at the 3, 6 and 12-month visits as part of this study, will be provided at no charge to you. Your GP will be compensated for the additional time it takes for them to conduct this research.

What are the possible benefits of taking part?

We cannot guarantee or promise benefits for you. However, possible benefits may include a quicker diagnosis and understanding of the cause of your breathlessness, more targeted tests and referrals, and advice about best management.

What are the alternatives to taking part in the research?

If you choose not to participate, you will still receive the usual level of medical care you otherwise would expect.

What will happen to my information?

- The answers to questions that you submit and your relevant health data from the GP electronic medical notes will be de-identified and securely stored on a computer server at The George Institute for Global Health that is password restricted. Only authorised research team members will see the data (which cannot identify you) and analyse it at the end of the trial.
- Your information will be stored for 15 years, in compliance with National Health and Medical Research (NHMRC) requirements
- We will keep your information private by assigning your data a unique study identifier before extracting it and not using your name on any document or data file.

Consent – Services Australia

You will be asked to read and sign a separate consent form authorising the BREATHE study to access your health information provided by Services Australia, we will send you a separate Services Australia Participant Information Document and Participant Consent Form after you see the GP at the 3mo visit. We would like you to read this document and decide if you agree that your health data can be made available to assist the researchers to understand what other medical services you have accessed in the 12 months you participate in the BREATHE project e.g. medications or specialist visit.
Services Australia is not involved in the BREATHE study, other than to provide the information that you have consented to the release of, should you decide to participate in this study. Services Australia has confirmed that this research and any associated documents have received approval from a Human Research Ethics Committee (HREC) that is registered with and operates within the guidelines set out by the National Health and Medical Research Council (NHMRC). Not consenting to this component of the trial will not affect your participation in BREATHE.

Improving the accuracy and efficiency of assessing and managing patients with breathlessness in primary care:

BREATHE, a real-world open label, cluster-randomised controlled trial

BREATHE
The Breathlessness Rapid Evaluation And THErapy study

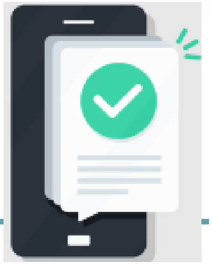

How and when will I find out what the results of the research study are?
At trial completion, we will provide copies of the published results to you, as well as a plain language statement to explain the trial results more generically.

Ethics Committee Approval
Our research trial has ethics approval. This means it has been checked over to make sure we are doing it right and safely. The ethics committee that approved our research is the UNSW Human Research Ethics Committee (HREC). If you think we are not doing the research right or if you have any complaints, you can contact the HREC with this reference number: iRECS6645. Phone: +61 2 9385 6222 or Email: humanethics@unsw.edu.au

Questions or concerns?
You can always speak with your GP about the research project, but if you would like to contact a study team member to discuss the study in more detail you can contact: Dr. Allison Humphries Email: ahumphries@georgeinstitute.org.au Telephone: +61 2 8052 4383 or Dr Katrina Giskes Email: katrina.giskes@nd.edu.au Telephone: +61 2 8204 4134

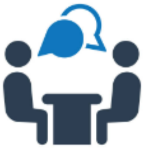

Supplement: online supplemental file 4 [file bmjopen-15-12-s004.pdf]
