# Supplementary material for: Digital support for chronic dyspnoea management in primary care: protocol for the BREATHE (Breathlessness Rapid Evaluation and Therapy) cluster randomised controlled trial
Source: BMJ Open. 2025 Dec 31;15(12):e108255. doi: 10.1136/bmjopen-2025-108255 (PMC13059914; doi:10.1136/bmjopen-2025-108255)
Supplement: Supplementary file 5 [file bmjopen-2025-108255.reviewer_comments.pdf]

PEER REVIEW HISTORY

BMJ Open publishes all reviews undertaken for accepted manuscripts. Reviewers are asked to complete a checklist review form and are provided with free text boxes to elaborate on their assessment. These free text comments are reproduced below.

ARTICLE DETAILS

Title (Provisional)

Digital Support for Chronic Dyspnea Management in Primary Care: Protocol for the BREATHE (Breathlessness Rapid Evaluation and Therapy) Cluster Randomised Controlled Trial

Authors

Martin, Allison; Sunjaya, Anthony Paulo; Giskes, Katrina; McKeough, Zoe; Hespe, Charlotte Mary; Arnott, Clare; Billot, Laurent; Campain, Anna; Scowcroft, Caroline Polak; Atkins, Emily; Jan, Stephen; Scott, Hayley; Chua, Ai-Vee; Jenkins, Christine Russell; Investigators, BREATHE

VERSION 1 - REVIEW

**Reviewer**	**1**
**Name**	**Peric, Ratko**
**Affiliation**	**OrthoSport, Exercise Physiology**
**Date**	**30-Sep-2025**
**COI**	**None**

This is a well-written and comprehensively detailed protocol for a pragmatic, cluster-randomised controlled trial (cRCT). The study addresses a significant and prevalent clinical problem—the under-diagnosis and suboptimal management of chronic dyspnea in primary care—with an innovative digital health solution. The protocol demonstrates a strong design, appropriate outcome measures, and a clear plan for implementation and analysis. The integration with existing practice software (BetterConsult/Best Practice) is a major strength for real-world applicability.

The manuscript is generally clear but would benefit from minor restructuring for improved flow and the correction of several typographical and grammatical errors. Please find my comments further below.

Title, Abstract, and Article Summary

The limitation stated is that the CDSS "only integrates with best practice medical software at present." This is accurate but could be framed more positively. Would suggest rephrasing to:

"A current limitation is the integration with a single practice software system (Best Practice); however, this serves as a proof-of-concept, with future work planned for broader implementation.

"Eligible patients will be ≥ 18 years old and previously consented to SMS communication..." is awkward phrasing. Change to "Eligible patients will be ≥18 years old and will have previously consented to receive SMS communication from their practice.

The hypothesis could be more specific and measurable. Refine the hypothesis to something similar to "We hypothesize that the use of the CDSS will lead to a clinically significant improvement in patient-reported dyspnea scores at 12 months compared to standard care."

Specify the primary time point for the primary outcome measurement for clarity. Maybe rephrase to: "The primary outcome is the improvement in the Dyspnea-12 (D-12) score from baseline to 12 months."

Introduction

The description of the partnership with the BREATHE SMART project is slightly confusing, as it is initially presented as a separate entity. Clarify the relationship from the outset. State clearly that the BREATHE trial incorporates the BREATHE SMART screening tool as the primary mechanism for identifying eligible participants. This avoids the perception of a separate, loosely linked study.

The statement "there is significant potential to support GPs..." could be strengthened. Immediately follow this statement by citing evidence of the current, specific gaps in dyspnea screening and risk stratification that the CDSS aims to fill.

Methods and Analysis

The eligibility criterion "Consent to SMS communication with their GP practice" may introduce a selection bias, potentially excluding older or less technologically adept populations who bear a significant burden of chronic dyspnea. Acknowledge this potential limitation in the Discussion section and briefly describe any strategies to mitigate it (e.g., offering practice staff assistance for patients to complete the screening in the waiting room).

The "Data Model" and "Knowledge Model" columns are not fully explained in the text. Add a footnote to the table explaining the meaning of "YES" and a blank cell. For example: "YES" indicates the diagnosis was included based on derivation from that model. A blank cell indicates it was not part of that model's derivation.

The description of the CDSS algorithm lacks specific functional details crucial for scientific appraisal. Provide a brief, high-level summary of how the algorithm works. For example: "The CDSS algorithm uses a Bayesian framework to calculate posterior probabilities for each of the 22 potential diagnoses based on the integrated input of patient demographics, pre-screening symptom data, GP-entered physical examination findings, and historical data extracted from the eMR.

The list of secondary outcomes is comprehensive, but the thresholds for "disease control" or "clinically important improvement" for each questionnaire (CAT, KCCQ, etc.) are not defined. Define the specific thresholds (e.g., "a reduction in CAT score of ≥2 points") in this section or explicitly state that these definitions will be detailed in the full Statistical Analysis Plan (SAP).

It would be beneficial to add details on data quality assurance. Add a sentence clarifying procedures for ensuring data quality, such as automated logic checks on data entry, protocols for flagging missing data, and procedures for querying sites.

Statistical Analyses

The assumption of a 10% dropout rate should be justified, especially for a 12-month follow-up in a primary care population with chronic conditions. Briefly justify the 10% dropout assumption, citing similar primary care trials or pilot data if available.

Even though the primary analysis plan is appropriate, strengthen this section by pre-specifying any planned sensitivity analyses (e.g., a per-protocol analysis) and explicitly stating the intended method for handling missing data (e.g., multiple imputation).

Ethics and Dissemination

The statement "No adverse events will be specifically collected" is potentially problematic. While the intervention itself is low-risk, its consequences (e.g., a missed diagnosis influenced by the CDSS) could be study-related. Revise this statement to clarify that the study will actively monitor for and record any serious adverse events (SAEs) that could be reasonably considered related to the study procedures. This includes diagnostic errors or significant treatment delays potentially attributable to the use or guidance of the CDSS.

**Reviewer**	**2**
**Name**	**Delclaux, Christophe**
**Affiliation**	**Université Paris Cité Faculté de Santé, Physiology**
**Date**	**10-Oct-2025**
**COI**	**None**

Major comment

I've difficulties with the endpoints of the study. The authors state "The objectives of this study are to assess the clinical impact of a CDSS for chronic dyspnea in primary care", which is a valuable aim. The authors also state that CDSS uses a validated and evidence-based dyspnea algorithm that may improve detection, diagnosis and management of the condition. When looking at the references provided (20, 21), the ref 20 deals with the Optimum Patient Care Research Database, without any specific reference to dyspnea, and the ref 21 is "under review". So it is difficult to affirm that this "CDSS uses a validated and evidence-based dyspnea algorithm". Finally, if this CDSS is validated, I guess that the algorithm may improve

diagnosis, which remains to be demonstrated, but whether the management of the condition will further be improved seems rather challenging.

Overall, I don't understand the primary objective: "The primary outcome is improvement in dyspnea over 12 months, measured by the Dyspnea- 12 (D-12) questionnaire". Some of the secondary objectives are easier to understand such as time to final diagnosis, and healthcare utilisation, costs...

Minor comment

Statistical analyses: "a drop-out rate of 10%, we aim to recruit 10 participants per cluster for a total of 400 participants" A drop-out rate of 10% is rather low in such a study implying multiple connections for both the GP and patient.

VERSION 1 - AUTHOR RESPONSE

Reviewer 1 Comments to Author

TITLE, ABSTRACT, SUMMARY	
The limitation stated is that the CDSS "only integrates with best practice medical software at present." This is accurate but could be framed more positively. Would suggest rephrasing to: "A current limitation is the integration with a single practice software system (Best Practice); however, this serves as a proof-of-concept, with future work planned for broader implementation.	Thank you for this suggestion. We have amended the limitation accordingly. However, we would like to note that Best Practice software is reported to be used in approximately 70% of GP practices, according to the PIPQO report from 31 Primary Health Networks (PHNs) in 2023-2024 (Practice Incentives Program Quality Improvement Measures, Australian Institute of Health and Welfare)
"Eligible patients will be ≥ 18 years old and previously consented to SMS communication..." is awkward phrasing. Change to "Eligible patients will be ≥18 years old and will have previously consented to receive SMS communication from their practice.	Thank you for this suggestion. We have amended the text accordingly.
The hypothesis could be more specific and measurable. Refine the hypothesis to something similar to "We hypothesize that the use of the CDSS will lead to a clinically significant improvement in patient-reported dyspnea scores at 12 months compared to standard care."	Thank you for the suggestion, we have amended the hypothesis to state "We hypothesise that the use of the CDSS will lead to a clinically significant improvement in patient-reported dyspnea scores, reduced time to diagnosis and healthcare costs at 12 months compared to standard care."
Specify the primary time point for the primary outcome measurement for clarity. Maybe rephrase to: "The primary outcome is the improvement in the Dyspnea-12 (D-12) score from baseline to 12 months."	Thank you for the suggestion. We have amended the text accordingly.

INTRODUCTION	
The description of the partnership with the BREATHE SMART project is slightly confusing, as it is initially presented as a separate entity. Clarify the relationship from the outset. State clearly that the BREATHE trial incorporates the BREATHE SMART screening tool as the primary mechanism for identifying eligible participants. This avoids the perception of a separate, loosely linked study.	Thank you for the comment. Additional detail and a Figure have been added to clarify the BREATHE SMART screening tool being used to identify eligible participants.
The statement "there is significant potential to support GPs..." could be strengthened. Immediately follow this statement by citing evidence of the current, specific gaps in dyspnea screening and risk stratification that the CDSS aims to fill.	Thank you for this suggestion. Additional information to strengthen this statement has been added in the revised Introduction. Specifically, the following statement highlights the significant gaps that this research addresses "An estimated 70% of people with asthma or COPD remain undiagnosed, and hence untreated in the community [3, 4]"
METHODS AND ANALYSIS	
The eligibility criterion "Consent to SMS communication with their GP practice" may introduce a selection bias, potentially excluding older or less technologically adept populations who bear a significant burden of chronic dyspnea. Acknowledge this potential limitation in the Discussion section and briefly describe any strategies to mitigate it (e.g., offering practice staff assistance for patients to complete the screening in the waiting room).	We have added this as a limitation in the "Strengths and Limitations" section of the Article Summary and in the Discussion section.
The "Data Model" and "Knowledge Model" columns are not fully explained in the text. Add a footnote to the table explaining the meaning of "YES" and a blank cell. For example: "YES" indicates the diagnosis was included based on derivation from that model. A blank cell indicates it was not part of that model's derivation.	Further detail is added in the text under the heading "Intervention" to explain the differences in Table 1 of the YES and blank cells, as well as amending the title of the table.
The description of the CDSS algorithm lacks specific functional details crucial for scientific appraisal. Provide a brief, high-level summary of how the algorithm works. For example: "The CDSS algorithm uses a Bayesian framework to calculate posterior probabilities for each of the 22 potential diagnoses based on the integrated input of patient demographics, pre-screening symptom data, GP-entered physical examination findings, and historical data extracted from the eMR.	This detail has been added to the text under the heading "Intervention"

The list of secondary outcomes is comprehensive, but the thresholds for "disease control" or "clinically important improvement" for each questionnaire (CAT, KCCQ, etc.) are not defined. Define the specific thresholds (e.g., "a reduction in CAT score of ≥2 points") in this section or explicitly state that these definitions will be detailed in the full Statistical Analysis Plan (SAP).	We have added the clinical thresholds for improvement in these questionnaires to the “Methods” section
It would be beneficial to add details on data quality assurance. Add a sentence clarifying procedures for ensuring data quality, such as automated logic checks on data entry, protocols for flagging missing data, and procedures for querying sites.	Additional detail on the data monitoring processes has been added to the “Data Collection” section
STATISTICAL ANALYSIS	
The assumption of a 10% dropout rate should be justified, especially for a 12-month follow-up in a primary care population with chronic conditions. Briefly justify the 10% dropout assumption, citing similar primary care trials or pilot data if available.	We have not found any published cluster randomised trials of a CDSS for diagnosis in primary care and therefore have been unable to accurately calculate a reasonable drop-out rate. Noting that our recruitment of 400 participants is the minimum number expected. Each of the 40 practices can recruit higher numbers if they choose to. The sample size is calculated on GP practice numbers, not participants. We considered 10% as a conventional assumption but will monitor recruitment closely.
Even though the primary analysis plan is appropriate, strengthen this section by pre-specifying any planned sensitivity analyses (e.g., a per-protocol analysis) and explicitly stating the intended method for handling missing data (e.g., multiple imputation).	Additional text has been added to the statistical analysis section detailing the sensitivity analysis and secondary outcomes.
ETHICS AND DISSEMINATION	
The statement "No adverse events will be specifically collected" is potentially problematic. While the intervention itself is low-risk, its consequences (e.g., a missed diagnosis influenced by the CDSS) could be study-related. Revise this statement to clarify that the study will actively monitor for and record any serious adverse events (SAEs) that could be reasonably considered related to the study procedures. This includes diagnostic errors or significant treatment delays potentially attributable to the use or guidance of the CDSS.	We appreciate the reviewer’s thoughtful feedback regarding the monitoring of adverse events. We have revised the statement to clarify that while the intervention is low-risk and designed to support clinical decision-making, we acknowledge the importance of monitoring for any serious adverse events that could reasonably be considered related to study procedures.<br><br>Clinicians retain full autonomy over all decisions regarding further investigations, management, and diagnosis. The CDSS is intended solely to provide non-binding

	suggestions, which clinicians may choose to accept or disregard at their discretion. It does not override clinical judgement or decision-making.<br><br>To ensure appropriate oversight, we have included in our training with participating GPs and practice managers that any issues, problems, adverse events, or patient complaints related to the research should be directed to the research team. Additionally, all participants (including practices, GPs, and patients) are provided with contact details for the relevant ethics committees in the participant information statements, should a serious adverse event or concern arise.<br><br>We have updated our manuscript to reflect that the study will actively monitor and record any serious adverse events that could reasonably be considered related to the study procedures, including diagnostic errors or significant treatment delays potentially attributable to the CDSS.

Reviewer 2 Comments to Author

I've difficulties with the endpoints of the study. The authors state "The objectives of this study are to assess the clinical impact of a CDSS for chronic dyspnea in primary care", which is a valuable aim. The authors also state that CDSS uses a validated and evidence-based dyspnea algorithm that may improve detection, diagnosis and management of the condition. When looking at the references provided (20, 21), the ref 20 deals with the Optimum Patient Care Research Database, without any specific reference to dyspnea, and the ref 21 is "under review". So it is difficult to affirm that this "CDSS uses a validated and evidence-based dyspnea algorithm". Finally, if this CDSS is validated, I guess that the algorithm may improve diagnosis, which remains to be demonstrated, but whether the management of the condition will further be improved seems rather challenging.	We agree that clearly defined endpoints are essential to evaluating the clinical impact of the CDSS.<br><br>In response to Part 1 of the Reviewer feedback, we have clarified the study endpoints in the revised manuscript. This details the primary and secondary outcomes that reflect both clinical outcomes (i.e. severity of dyspnea and its impact on patients) and health service utilisation outcomes (including diagnostic investigations, referrals, and follow-up care).<br><br>Together, these endpoints are designed to comprehensively assess the clinical utility of the CDSS in primary care settings, capturing both patient-centred and system-level impacts.<br><br>Part 2 of the Reviewer comment:<br><br>Publication of the validation study is currently underway; the referenced manuscript is currently under peer review and provides detailed evidence supporting the

	development and validation of the CDSS. To assist, additional information has been added to page 5 (Intervention) to describe more clearly the process undertaken for the algorithm development.<br><br>In addition to evaluating the algorithm’s clinical utility, a primary goal of this research is to test a proof-of-concept for the implementation of a fully integrated CDSS for dyspnea detection and management in general practice. This includes assessing how well the CDSS integrates into the GP workflow and supports preventive health capabilities in primary care settings.
Overall, I don't understand the primary objective: "The primary outcome is improvement in dyspnea over 12 months, measured by the Dyspnea- 12 (D-12) questionnaire". Some of the secondary objectives are easier to understand such as time to final diagnosis, and healthcare utilisation, costs.	We have amended the wording of the primary outcome to clarify further, it now reads: “The primary outcome is improvement in dyspnea, measured by a reduction of 2.83 points of the D-12 score at 3, 6 and 12 months”.
Statistical analyses: "a drop-out rate of 10%, we aim to recruit 10 participants per cluster for a total of 400 participants" A drop-out rate of 10% is rather low in such a study implying multiple connections for both the GP and patient.	This has been responded to above as Reviewer 1 had the same comment.
